# Comparative study of trans* healthcare models in Catalonia

**DOI:** 10.1016/j.heliyon.2024.e36174

**Published:** 2024-08-14

**Authors:** Maria Presague-Peciña, Pepita Giménez-Bonafé

**Affiliations:** a Faculty of Medicine and Life Sciences, Dr Aiguader Building, Pompeu Fabra University, 08003, Barcelona, Spain; b Department of Physiological Sciences, Physiology Unit, Faculty of Medicine and Health Sciences, Bellvitge Campus, Universitat de Barcelona, IDIBELL, 08907, L'Hospitalet del Llobregat, Barcelona, Spain

**Keywords:** Trans* health, Gender diversity, Biomedical model, Biopsychosocial model, Pathologization, Mental health

## Abstract

Stigma and discrimination against the trans* community have been shown to exacerbate mental health issues among its members. In Catalonia, the Gender Identity Unit at the Clinic's Hospital traditionally adhered to a biomedical model (BMM) of health for trans* individuals. However, a few years ago, the Transit Service introduced a biopsychosocial model (BPSM). This observational cohort study explores the mental health effects in BMM compared to BPSM centers for trans* individuals.

A web-based survey was employed to gather essential data, such as socio-demographics information, and mental health outcomes. All data was analyzed from the BPSM group (n = 81) and the BMM group (n = 21).

The BPSM group exhibited statistically significant lower odds of experiencing emotional distress (p < 0.001). Other mental health outcomes indicated a trend toward lower odds in the BPSM group compared to the BMM group. Nevertheless, the prevalence of mental health problems were much higher than expected in general popupation, both groups presented depression rates of 35 % and 25.7 %, and anxiety rates of 45 % and 41.9 % (BMM and BPSM, respectively). Furthermore, these differences were also found when comparing to general population in Catalonia.

Therefore, there is a pressing need to shift away from paternalistic medical roles and move towards informed decision-making and progressive autonomy. Perceiving the trans* experience as an individual desease, rather than an effect of societal norms on dissident bodies, has detrimental effects for the community. Additionally, the scientific community should listen to the demands of the trans* community and create space for trans* researchers in the production of knowledge.

## Highlights

• The trans* community has long been fighting against the pathologization of gender diversity and dissident corporalities for years. Transphobia, in particular has been linked to increased rates of anxiety, depression, and suicide.
• Our study indicates that adopting healthcare models that embrace an inclusive perspective on gender identity, could improve trans* community mental health rates.
• Healthcare centers shoud move away from paternalistic approaches and transition towards models that prioritize informed decision-making and autonomy.
• There is a pressing need for more scientific research to address the unique needs of the trans* community, with a strong emphasis on actively involving trans* researchers in the production of scientific knowledge.

**Table:** List of abbreviations

Abbreviations	Full writing
BMM	Biomedical Model
BPSM	Biopsychosocial Model
GIDSEEN group	Identity and Sexual Differentiation Group promoted by the Endocrinology and Nutrition Spanish Society (in Spanish: Grupo de Identidad y Diferenciación Sexual promoted by the Sociedad Española de Endocrinología y Nutrición).
GIU	Gender Identity Unit
LGTBIQ+	Lesbians, Gays, Trans, Bisexuals, Intersexuals, Queer, among others
WEMWBS	Warwick-Edinburgh Mental Well-Being Scale
WHO	World Health Organization

## 1 Introduction

A lot has been said about trans* individuals, but has society truly listened to their reality? In recent years, the trans* community has been emerging from the closet, reclaiming public spaces long denied to them. Throughout this document, the asterisk accompanying the term “trans*" serves as an umbrella, encompassing various identities such as transsexual, transgender, transvestite, gender fluid, non-binary, or agender people, among others. For a clearer understanding, Table 1 provides definitions for some key terms.

**Table 1:** Definitions of terms.

Term	Definition
*Cisgender*	People who self-identify with a gender that matches their assigned sex at birth.
*Gender*	A social, cultural, and psychological construct that traditionally defines the concepts of man and woman based on cultural roles. It conceptualizes self-identity and behavior into categories such as man, woman, and non-binary, among others.
*Gender Dysphoria*	Term commonly used in scientific publications to describe the discomfort or distress arising from the incongruence between a person's gender identity and their assigned sex at birth (whether socially, physically, or both).
*Gender Expression*	Refers to how individuals manifest their gender identity, typically through aspects such as dress code, behavior, or appearance. This expression is often shaped by societal expectations and cultural norms related to gender, varying across different socio-cultural contexts. It is important to note that some individuals may exhibit a gender expression that doesn't conform to traditional binary norms, adopting an androgynous presentation that transcends conventional gender identities.
*Gender Identity*	Individual self-perception of one's gender, wherein a person identifies as a woman, man, a blend of the two (gender fluid), none of them (non-binary), or embraces other non-normative gender identities.
*Gender Transition*	The process through which transgender individuals adjust and live in accordance with their self-identified gender, particularly within all aspects of their lives, with a focus on the social sphere.
*Gender Role*	Social rules and behaviors perceived as appropriate based on gender within a specific group or social system are commonly known as gender norms. These norms are established in accordance with the social constructs surrounding masculinity and femininity.
*Sex*	Biological and physical characteristics encompass chromosomal, hormonal, gonadal, and genital attributes. Typically, the sex assigned at birth is determined by the newborn's genital appearance. It is often assumed that the other components of sex align with the genital sex assignment.
*Sexual Orientation*	Pattern of sexual or romantic attraction to a specific group of people, irrespective of their gender identity or expression.
Trans*	Term that includes all that people self-identified with a gender different from the assigned sex at birth, or that express gender in a non-normative way. Includes transsexuals, transgenders, crossdressers, transvestites, non-binary, gender fluid, and agender, among others.
*Transsexual*	There are various ways individuals experience and express their transsexuality. Broadly, many transsexual individuals perceive a need for body transformation through hormonal or surgical treatments and identify within the binary categories of man or woman.
*Transgender*	They do not feel compelled to conform to a normative alignment between sex and gender through complete body transformation. Some individuals question the necessity of adhering strictly to traditional male and female roles.
*Transphobia*	Fear and/or hatred directed towards trans people often manifested as discrimination, harassment, and violence against the trans community. The consequences of transphobia can span from psychological mistreatment and social exclusion to instances of severe harm or even death.
Trans* pathologizing	The process by which transsexuality is categorized as a mental disorder that can be diagnosed and treated through psychiatric methods.

The binary sex/gender system presumes that men and women are the only gender options. However, this lets many individuals out of the equation. Newborns are assigned a gender identity primarily based on their genitalia, influencing their behaviors and expressions, and offering little room for choice [1]. This assumption means that an individual's biological sex has to align with their gender identity or expression. Nevertheless, skepticism about the consistency of gender and sex binarism has existed for years. Anne Fausto-Sterling discussed these topics as early as in 1990 [2].

The trans* community has endured, and continues to face, the consequences of a harsh cultural system. As a result, trans* individuals experience discrimination, violence, human rights violations, and, in the worst cases, homicide [3]. Standardized notions of masculinity and femininity subject them to social stigma, often permeating scientific research [4]. The healthcare system bears a share of responsibility for this matter. According to an International Amnesty survey, as many as 30 % of trans* respondents encountered situations where healthcare providers, though willing to assist, lacked understanding of trans* issues [5].

Biomedical models (BMM) of healthcare have dominated industrialized societies since the mid-20th century, considering the biological basis of disease but often neglecting other factors influencing health outcomes. The biopsychosocial model (BPSM), proposed by George Engel, responded to this limitation, advocating for clinicians to address the biological, psychological, and social dimensions of patients' health simultaneously [6]. Borrell-Carrió F et al. emphasized the patient-clinician relationship, suggesting a change in the patient role, going from being a passive object of investigation to a subject of the clinical act [7].

Since its inception, the medical approach to trans* healthcare has been critiqued from a perspective rooted in trans* activism and theory [3]. For a better understanding of the historical background of trans* healthcare models in Spain, refer to Appendix I.

Initially, Spanish institutions adopted the Biomedical model (BMM) of healthcare through Gender Identity Units (GIUs). They were criticized for losing sight of gender self-determination and focusing on the diagnosis and treatment of gender dysphoria. These centers operate under GIDSEEN group guidelines, establishing a standard itinerary for trans* individuals [8]. Initially, individuals were directed to a psychiatrist consultation and then had to undergo the *Real-Life Test*, requiring them to socialize and adjust their image to the “desired” gender for at least three months. They were expected to maintain their jobs or academic results with a “high level of satisfaction” to access hormone and surgical therapies [[8], [9], [10]].

Following classical gender roles, surgical treatment was considered essential. This was not a coincidental phenomenon, but rather a response to the imperative need to maintain a population within the hegemonic binarism. The collective mindset was not yet prepared to accept the true diversity of trans* people. Therefore, the stereotyped “true transexual” was gradually being formed [9].

However, in more recent times, there has been an increasing recognition of self-identification, self-acceptance, and social visibility as unique processes for each individual [11,12]. The Transit Service emerged as a Biopsychosocial model (BPSM) of trans* healthcare, emphasizing autonomy and informed consent. They provide information and counseling tailored to individual needs, creating welcoming and inclusive environments. All decisions rest with the individual, without the need for diagnosis [1,13,14].

Discrimination against trans* individuals can lead to emotional distress and mental health issues. Dhejne et al. found that mental health disorders appeared to be more prevalent among the trans* population compared to cisgender controls [15]. Additionally, for non-binary youth, receiving gender-affirming care is associated with a 60 % reduction in moderate to severe depression [16]. Zeluf et al. examined the prevalence of suicide ideation in Sweden and found that trans* individuals were seven and a half times more likely to have suicidal thoughts than cisgender individuals [17]. This study also revealed a link between transphobic treatment and suicide.

Therefore, the aim of the study is to fullfill the lack of investigations comparing BMM and BPSM healthcare centers on trans* individuals and highlight the need of more research about these topics. Additionally, rates of healthcare center evaluations, deception in consultation, and self-treatment (hormonal therapies without medical supervision) will be analyzed as secondary objectives.

## 2 Materials and methods

### 2.1 Study design and participants

This observational cohort study compares two distinct groups: trans* individuals who accessed the BMM (represented by Clinic Hospital's GIU) and trans* individuals who accessed the BPSM (represented by Transit Service). We designed a web-based open survey, in collaboration with ACATHI (migrant LGTBIQ+ association in Catalonia), Chrysallis Catalunya (transgender children and youth association), and Casal Lambda (an association within the LGTBIQ+ Barcelona Center that promotes psychological and legal counseling, as well as support services for the LGTBIQ+ community). The final survey can be found on Supplementary data.

The survey form was not statistically analyzed, and was shared to confirm proper understanding of all questions. Additionally, LGBT organizations that participated proposed improvements in the questions that were asked.

To ensure robust results, we adhered to established survey research methodology, including an introduction outlining the research purpose, researcher identification, and contact information [18]. Furthermore, we applied the Checklist for Reporting Results of Internet E-Surveys (CHERRIES) [19]. The survey was tested with various individuals both inside and outside the scientific community. Therefore, the accurate comprehension of the questions was thoroughly revised. Concretely the study was reviewed by the LGTBIQ+ organizations themselves and scientific researchers from other institutions, who gave their opinion on the matter and proposed changes.

### 2.2 Study population

Participants responded to the survey from November 2022 to February 2023. The inclusion criteria involved self-identifying as a transgender individual and accessing at least one of the two healthcare centers under investigation. Exclusion criteria encompassed individuals who sought healthcare outside Catalonia and those not identifying as transgender. To comprehend the reasons for non-accessing, individuals who did not access any transgender healthcare center were also analyzed.

The two healthcare centers were not mutually exclusive, resulting in data collection for four distinct groups: the BPSM group, the BMM group, the BMM and BPSM group, and the non-access group.

### 2.3 Survey and variables of study

The survey encompassed participants' general information, including age, gender identity and expression, sexual orientation, biological sex. Age was treated as an ordinal qualitative variable, as the participants were not asked for the specific age. The other variables were not treated as binary variables. In addition, participants were asked about social determinants of health, such as being a trans* women, trans* youth, trans* elderly, or migrated individuals, among others. The participants' mental health history was also documented. Indeed, these variables were treated as binary (Yes/No answer). The subjective well-being of participants was measured using the World Health Organization's 5-question Well-Being Index [20]. This was the only quantitative variable. For further information, the survey can be reviewed in Supplementary Data.

Information related to the healthcare center included mental health outcomes during the transition and a general assessment of the care received. Participants were also queried about self-treatment practices (before, during, or after accessing the center), psychiatric and endocrinologic attention, as well as surgery interventions.

### 2.4 Data collection and participants recruitment

To ensure an adequate number of participants, the study survey was distributed through all trans* associations in Catalonia. The organizations were approached to share the survey among their members. Both Transit and the GIU of Clinic's Hospital administered the questionnaire to individuals attending their respective centers. Throughout the study, a designated contact address was provided for participants to solve any questions they might have had. All willing participants were included in the study. Anonymous volunteers responded the form.

Additionally, a dedicated webpage (https://mariapres21.wixsite.com/estudi-comparatiu-de) was established to detail the study's information. The participation of all organizations involved in survey distribution is duly acknowledged in this publication.

Originally, a modified survey was crafted to facilitate responses from youth under parental supervision. This adapted survey employed clear and concise language, featuring a reduced set of questions. Chrysallis Catalunya played a crucial role in distributing the survey to the parents of trans* youth. Consequently, minors up to the age of 16 participated with the consent of their parents.

### 2.5 Data analyses

Descriptive analyses were employed to furnish comprehensive information on the demographic characteristics of study participants. The Chi-square test, or Fisher's exact test when necessary, was used to discern associations in mental health outcomes within both groups. Qualitative variables were presented as frequencies and percentages (%). Mean and standard deviation were employed for quantitative variables, and these were analyzed using Student's T-test. Statistical significance was defined as p ≤ 0.05, and all analyses were conducted as two-sided tests. IBM SPSS 25.0 (Chicago, IL, USA) was the statistical software used for all analyses.

## 3 Results

**Fig. 1:**
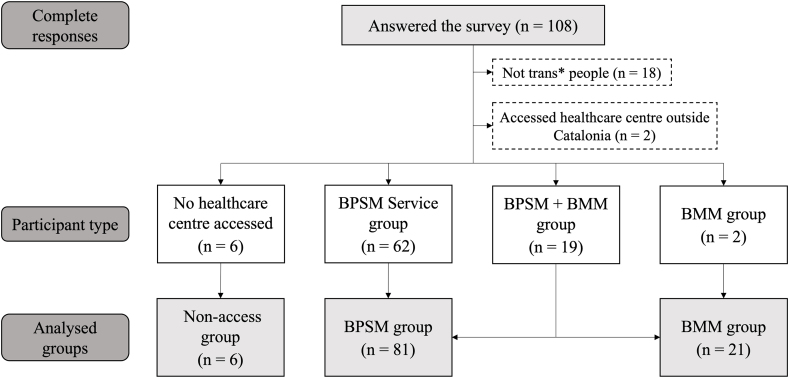
Flow chart of survey participants. BPSM: Biopsychosocial model; BMM: Biomedical model.

The survey garnered responses from 108 trans* individuals (Fig. 1). Twenty participants were excluded for failing to meet the inclusion criteria. Among the respondents, nineteen had accessed both healthcare centers and were categorized within the BMM group for sociodemographic analysis. However, for healthcare center analysis, they were further divided into the BMM and BPSM groups. Six participants who did not utilize any healthcare center were excluded from the analysis, although they are discussed later. Regrettably, there were no responses to the youth survey.

The final sample comprised the BPSM group (n = 81) and the BMM group (n = 21). Social-demographic characteristics are detailed in Table 2. Mental health history rates were substantial, with 81 % and 65.6 % in the BMM and BPSM groups, respectively (Table 2).

**Table 2:** Social-demographic characteristics of study participants.

*Characteristic*	*BMM Group*		*BPSM Group*		*P-values*
	*n = 21 (%)*		*n = 62 (%)*		
Age (years)					
16–18	2	(9.5)	4	(6.5)	p = 0.100
18–20	1	(4.8)	6	(9.7)	
20–25	3	(14.3)	18	(29.0)	
25–30	2	(9.5)	13	(21.0)	
30–35	2	(9.5)	10	(16.1)	
35–40	2	(9.5)	1	(1.6)	
40–45	3	(14.3)	3	(4.8)	
45–50	2	(9.5)	2	(3.2)	
50–55	0	(0.0)	1	(1.6)	
55–60	4	(19.0)	2	(3.2)	
60–65	0	(0.0)	1	(1.6)	
> 65	0	(0.0)	1	(1.6)	
Gender Identity					
Women	8	(38.1)	15	(24.2)	p = 0.750
Man	6	(28.6)	22	(35.5)	
Non-binary	5	(23.8)	19	(30.6)	
Agender	1	(4.8)	4	(6.5)	
Gender Fluid	1	(4.8)	2	(3.2)	
Gender Expression					
Feminine	2	(9.5)	4	(6.5)	p = 0.495
Near – feminine	4	(19.0)	11	(17.7)	
Androgynous	1	(4.8)	10	(16.1)	
Near – masculine	14	(66.7)	30	(48.4)	
Masculine	0	(0.0)	5	(8.1)	
Missing	0	(0.0)	2	(3.2)	
Assigned sex at birth					
Woman	10	(47.6)	42	(67.7)	p = 0.194
Man	11	(52.4)	19	(30.6)	
Intersex	0	(0.0)	1	(1.6)	
Sexual Orientation					
Heterosexual	6	(28.6)	9	(14.5)	p = 0.432
Homosexual	2	(9.5)	4	(6.5)	
Bisexual	12	(57.1)	45	(72.6)	
Asexual	1	(4.8)	4	(6.5)	
Vulnerable Sectors^a^	19	(90.5)	52	(83.9)	p = 0.721
Trans* Underage	2	(9.5)	5	(8.1)	p = 1.000
Trans* Elder	4	(19.0)	4	(6.5)	p = 0.107
Women	10	(47.6)	19	(30.6)	p = 0.159
Low economic status	3	(14.3)	10	(16.1)	p = 1.000
No legal transition	2	(9.5)	20	(32.3)	**p = 0.041**
Trans* parents	0	(0.0)	3	(4.8)	p = 0.568
Migrated	1	(4.8)	6	(9.7)	p = 0.673
Deprived of liberty	0	(0.0)	0	(0.0)	
Sex workers	1	(4.8)	2	(3.2)	p = 1.000
HIV+	1	(4.8)	0	(0.0)	p = 0.253
Functional diversity	3	(14.3)	3	(4.8)	p = 0.167
Mental Health History^a^^,^^b^	17	(81.0)	40	(65.6)	p = 0.067
Depression	8	(38.1)	27	(44.3)	p = 0.622
Anxiety	15	(71.4)	32	(52.5)	p = 0.130
Eating Disorder	2	(9.5)	11	(18.0)	p = 0.498
Addictive Disorder	4	(19.0)	6	(9.8)	p = 0.269
Attempted Suicide	5	(23.8)	11	(18.0)	p = 0.541
Thoughts of death	10	(47.6)	20	(32.8)	p = 0.224
Self-harm	5	(23.8)	10	(16.4)	p = 0.516
ASD	5	(23.8)	10	(16.4)	p = 0.516
Personality Disorder	1	(4.8)	6	(9.8)	p = 0.671
Paranoid Disorder	1	(4.8)	1	(1.6)	p = 0.449
Schizophrenia	0	(0.0)	1	(1.6)	p = 1.000
Bipolar disorder	1	(4.8)	0	(0.0)	p = 0.256
Dementia	0	(0.0)	0	(0.0)	
OCD	2	(9.5)	5	(8.2)	p = 1.000
PTSD	1	(4.8)	13	(21.3)	p = 0.102
Emotional Distress (without diagnosis)	2	(9.5)	2	(3.3)	p = 0.270
Psychological care					
No	3	(14.3)	8	(12.9)	p = 0.936
Public services	4	(28.6)	20	(32.3)	
LGTBIQ + association	2	(9.5)	4	(6.5)	
Private services	10	(47.6)	30	(48.4)	
WHO5 Well Being Index	43.80 ± 20.66		50.71 ± 19.91		p = 0.185

ASD: Autism Spectrum Disorder; OCD: Obsessive-Compulsive Disorder; PTSD: Post-Traumatic Stress Disorder.

a These questions in the survey were designed as multiple-answer questions, allowing participants to select more than one option simultaneously. The first row in the results describes whether or not participants had at least one of the variables listed, indicating the presence of multiple diagnoses or conditions among respondents.

b This variable explains the mental health history, unlike further mental health variables.

### 3.1 Mental health outcomes

Both groups exhibited a significant proportion of mental health pathology, with approximately 50 % of individuals in each group reporting at least one mental health diagnosis during their healthcare center visits. Depression was reported by 35 % of respondents in the BMM group, and 25.7 % in the BPSM group. Anxiety was also reported by 45 % and 41 % of participants in the BMM, and BPSM groups, respectively. Additional associations with mental health outcomes are described in Table 3.

**Table 3:** Mental Health outcomes during healthcare centers visits.

*Mental Health Outcomes*	*BMM Group*		*BPSM Group*		*Odds Ratio*^a^*(95 % CI)*	*P - value*
	*n = 21 (%)*		*n = 81 (%)*			
Mental Health Variables^b^	10	(50.0)	36	(48.6)		p = 0.915
Depression	7	(35.0)	19	(25.7)	0.64 (0.22–1.84)	p = 0.408
Anxiety	9	(45.0)	31	(41.9)	0.88 (0.33–2.38)	p = 0.803
Eating Disorder	1	(5.0)	5	(6.8)	1.38 (0.15–12.50)	p = 1.000
Addictive Disorder	1	(5.0)	5	(6.8)	1.38 (0.15–12.50)	p = 1.000
Attempted Suicide	2	(10.0)	4	(5.4)	0.51 (0.09–3.03)	p = 0.604
Thoughts of death	5	(25.0)	10	(13.5)	0.47 (0.14–1.57)	p = 0.299
Self-harm	3	(15.0)	8	(10.8)	0.69 (0.16–2.87)	p = 0.696
Emotional Distress (without a diagnosis)	8	(42.1)	13	(17.6)	0.29 (0.10–0.87)	**p = 0.032**
I do not know/Missing^c^	0		7			

a Odds Ratio is referred to BPSM group.

b This variable indicates whether participants received a mental health diagnosis during their transition. Unlike the mental health variable presented in Table 2, this was a multiple-answer question, allowing participants to report more than one diagnosis. The first row represents individuals who received at least one diagnosis.

c Missing values for mental health outcomes were categorized under the “I do not know” group for inclusion in the table. It is important to note that these values were not considered in the analysis, but they are presented in the table for transparency and completeness of reporting.

The BPSM group was significantly associated with lower emotional distress (Odds Ratio [OR] 0.29; 95 % CI 0.10–0.87) (p < 0.05). Although other mental health outcomes did not show significant associations, they demonstrated a trend towards lower odds in depression (OR 0.64; 95 % CI 0.22–1.84), anxiety (OR 0.88; 95 % CI 0.33–2.38), suicide attempts (OR 0.51; 95 % CI 0.09–3.03), thoughts of death (OR 0.47; 95 % CI 0.14–1.57), and self-harm (OR 0.69; 95 % CI 0.16–2.87). In contrast, eating disorders and addictive disorders were associated with higher odds (OR 1.38; 95 % CI 0.15–12.50 for both variables). Nevertheless, these last two results should be interpreted cautiously due to the small sample size and wide confidence intervals limiting interpretability and generalizability.

### 3.2 Healthcare center evaluation

Participants were instructed to utilize a 5-point Likert scale to assess seven dimensions of healthcare centers. The findings for all dimensions were statistically significant (p < 0.001) and are outlined in Table 4.

**Table 4:** Healthcare center evaluation.

*Healthcare center evaluation*	*BMM Group*		*BPSM Group*		*P - value*
	*n = 21 (%)*		*n = 81 (%)*		
I was respected in my identity construction process					
Strongly agree	3	(14.3)	56	(75.7)	**p** < **0.001**
Agree	3	(14.3)	15	(20.3)	
Neutral	5	(23.8)	2	(2.7)	
Disagree	1	(4.8)	1	(1.4)	
Strongly Disagree	9	(42.9)	0	(0.0)	
Missing	0		7		
I felt questioned					
Strongly agree	9	(45.0)	1	(1.4)	**p** < **0.001**
Agree	2	(10.0)	1	(1.4)	
Neutral	4	(20.0)	6	(8.2)	
Disagree	3	(15.0)	14	(19.2)	
Strongly Disagree	2	(10.0)	51	(69.9)	
Missing	1		8		
I felt sick					
Strongly agree	4	(20.0)	0	(0.0)	**p** < **0.001**
Agree	6	(30.0)	0	(0.0)	
Neutral	3	(15.0)	8	(11.4)	
Disagree	5	(25.0)	7	(10.0)	
Strongly Disagree	2	(10.0)	55	(78.6)	
Missing	1		11		
My well-being was deteriorated					
Strongly agree	6	(30.0)	1	(1.4)	**p** < **0.001**
Agree	4	(20.0)	3	(4.1)	
Neutral	6	(30.0)	4	(5.5)	
Disagree	1	(5.0)	7	(9.6)	
Strongly Disagree	3	(15.0)	58	(70.5)	
Massing	1		8		
Classic gender roles were used several times					
Strongly agree	11	(55.0)	0	(0.0)	**p** < **0.001**
Agree	2	(10.0)	1	(1.4)	
Neutral	3	(15.0)	7	(9.7)	
Disagree	1	(5.0)	9	(12.5)	
Strongly Disagree	3	(15.0)	55	(76.4)	
Missing	1		9		
Healthcare workers empathized with me					
Strongly agree	4	(20.0)	53	(72.6)	**p** < **0.001**
Agree	2	(10.0)	15	(20.5)	
Neutral	5	(24.0)	3	(4.1)	
Disagree	2	(10.0)	2	(2.7)	
Strongly Disagree	7	(35.0)	0	(0.0)	
Missing	1		8		
I had a decision-making capacity about the treatment					
Strongly agree	2	(10.0)	58	(79.5)	**p** < **0.001**
Agree	5	(25.0)	12	(16.4)	
Neutral	1	(5.0)	2	(2.7)	
Disagree	5	(25.0)	1	(1.4)	
Strongly Disagree	7	(35.0)	0	(0.0)	
Missing	1		8		

^a^ Missing values were not counted in the analysis, however, they are shown in the table.

Overall, respondents in the BPSM group reported higher levels of satisfaction. Specifically, they were more likely to *strongly agree* and *agree* that their identity construction was respected (20.3 % and 75.7 %, respectively), and that they had decision-making capacity regarding treatment (16.4 % and 79.5 %, respectively).

Furthermore, approximately 50 % of BMM respondents *agreed* and *strongly agreed* that they felt both questioned and sick. Additionally, 10 % and 55 % of BMM respondents *agreed* and *strongly agreed*, respectively, that classical gender roles were used several times, while no one in the BPSM group strongly agreed with this, and only 1.4 % agreed.

### 3.3 Other variables

The study revealed a significantly higher proportion of respondents in the BMM group (40 %) admitting to lying in consultation, compared to the BPSM group (8.1 %) (p < 0.005). About 20 % of respondents in both groups reported engaging in self-treatment before accessing the healthcare centers (Table 5). However, a higher proportion of participants in the BMM group (9.5 %) reported self-treatment after accessing the center compared to the BPSM group (0 %) (p = 0.086).

**Table 5:** Lies in consultation, self-treatment and psychiatric and endocrinologic attention.

*Variables*	*BMM Group*		*BPSM Group*		*P - value*
	*n = 21 (%)*		*n = 81 (%)*		
Lies in consultation					
No	12	(60.0)	68	(91.9)	**p = 0.002**
Yes	8	(40.0)	6	(8.1)	
Missing	0		7		
Self-treatment					
No	14	(66.7)	61	(75.3)	p = 0.086
Yes, before accessing the center	5	(23.8)	17	(21.0)	
Yes, during the center access	0	(0.0)	3	(3.7)	
Yes, after accessing the center	2	(9.5)	0	(0.0)	
Psychiatric attention useful					
No	16	(76.2)	8	(23.5)	**p** < **0.001**
Yes	5	(23.8)	26	(76.5)	
Did not use/Missing^a^	0		47		
Endocrinologic attention useful					
No	11	(55.0)	4	(7.4)	**p** < **0.001**
Yes	9	(45.0)	50	(92.6)	
Did not used/Missing^a^	1		27		
Surgery					
No, but I will in the future	5	(23.8)	15	(20.0)	p = 0.074
No, I do not want them	9	(42.9)	14	(18.7)	
No, I did it on private healthcare	4	(19.0)	18	(24.0)	
Yes	3	(14.3)	28	(37.3)	
Missing	0		6		
Surgery Cost (if private) (€)	6375 ± 750		7683 ± 4206		p = 0.550

a ^”^Missing” and “Did not use” values were not included in the analysis; nevertheless, they are presented in the table for transparency and completeness of reporting.

A significantly higher proportion of BPSM group participants reported finding psychiatric attention useful (76.5 %) compared to the BMM group (23.8 %) (p < 0.001). Regarding endocrinologic attention, 92.6 % of BPSM participants reported finding it useful compared to 45 % of the BMM group (p < 0.001).

Surgical necessities varied, but a higher proportion of respondents in the BMM group (42 %) reported not wanting surgeries compared to the BPSM group (18 %) (p = 0.074). Furthermore, 19 % of BMM respondents accessed private surgical care (spending 6375 ± 750 euros) compared to 24 % of BPSM respondents (spending 7683 ± 4206 euros). Although private surgical services were external to the healthcare centers studied, this variable was included to gain insight into the costs incurred by trans* people who require such surgeries.

Six participants were ultimately not analyzed in comparison to other groups*,* because they did not access any trans* healthcare center. They never engaged in self-treatment or needed surgeries. Among this group, 60 % did not know why they did not access trans* healthcare centers, 20 % did not need it, and 20 % did not notice the centers*.*

## 4 Discussion

**Fig. 2:**
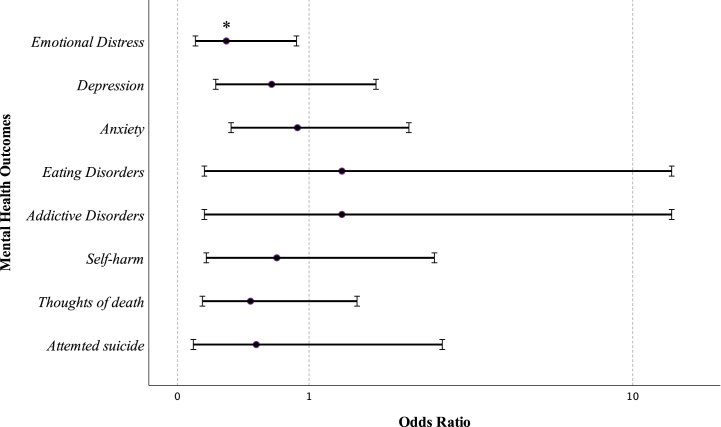
Odds Ratio plot for mental health outcomes in BPSM. Odds ratio axis is represented in logarithmical scale. *p < 0.05. BPSM: Biopsychosocial model; BMM: Biomedical model.

The study uncovered that individuals receiving care at BPSM healthcare centers had lower odds of experiencing emotional distress (p < 0.05), supporting the hypothesis that BMM may overly emphasize medical aspects and neglect the psychosocial aspects of trans* people. Although other mental health outcomes were not statistically significant, there was a trend towards lower odds in BPSM for mental health outcomes (Fig. 2). The BMM's assumption that trans* identities are a condition to be diagnosed and treated might increase psychological pressure, resulting in poor mental health outcomes [15]. These issues had been already discussed by trans* community in 2016 [14].

The two groups under study had similar socio-demographic characteristics (Table 2). Yet, trans* individuals without legal transition were more frequent in the BPSM group, potentially overestimating the prevalence of mental health issues. Trans* people without legal transition face more challenges in administrative processes, such as education, housing, or the labor market, which might affect their mental health outcomes [14].

The number of participants with a history of mental health conditions was notably high (Table 2), raising significant concern. For instance, individuals who had previously attempted suicide accounted for 23.8 % in the BMM group and 18 % in the BPSM group. To gain deeper insight into the implications of mental health disorders among trans* individuals, a comparison with the general population in Catalonia in 2021 was performed. Data from the Catalonia Health Survey (ESCA, in Catalan) was used, a survey conducted annually [21].

The ESCA survey collects data on health status, health-related behaviors, and health services use, including questions about depression, anxiety, and suicidal thoughts and attempts. Additionally, the Warwick-Edinburgh Mental Well-Being Scale (WEMWBS) is used to evaluate emotional distress [22]. Furthermore, the Barcelona Health Survey specifically asks participants about suicidal thoughts and attempts [23]. Approximately 85 % of the study participants accessed healthcare centers in Barcelona. Although the method of assessment differed from that used in these surveys, the comparison illustrated that the trans* community is at risk due to their dissident gender identity.

**Fig. 3:**
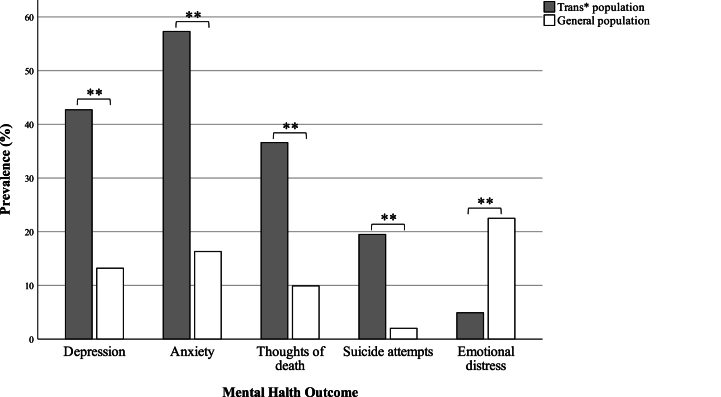
Bar chart of mental health outcomes in the study trans* population compared to Catalonia general population. Trans* population data was extracted from the present study. General population depression, anxiety and emotional distress data were extracted from the Catalonia Health Survey. General population thoughts of death and suicide attempts were extracted from the Barcelona Health Survey. **p < 0.001.

The results demonstrated a substantial difference in the prevalence of mental health issues between the trans* community in the study and the general population in Catalonia (Fig. 3). These findings underline the urgent need to implement effective public policies to protect trans* individuals and human rights. Emotional distress rates were the only aspect lower in the trans* community, potentially due to differences in data collection between surveys. Recent events, such as the COVID-19 pandemic or economic crisis, might have increased rates of mental health diagnosis, potentially changing people's status from emotional distress to more severe disorders compared to the 2021 Catalan population. Goldberg et al. found associations between COVID-19 pandemic lockdown stressors and depression and anxiety [24].

The call for further research to understand the social determinants of health for trans* individuals, considering the effects of gender identity dissidence along with other forms of exclusion or discrimination (e.g., racism, colonialism, classism, or ableism), is crucial, as highlighted by Veale et al. [25].

The study unveiled elevated rates of dishonesty during consultations in the BMM group compared to the BPSM group, with reports of individuals creating false information to secure hormonal treatments in Spanish Gender Identity Units (GIUs) [9,14]. This contradicts the notion of diagnosing the “authentic transsexual” and implies that trans identities encompass a broader spectrum than traditional gender roles suggest. Additionally, individuals who genuinely do not meet the diagnostic criteria in the BMM approach may face barriers accessing to hormone therapy, potentially resulting in increased cases of self-treatment within the community.

Significant differences in self-treatment rates after accessing healthcare centers were found, with no one engaging in self-treatment in BPSM, while 9.5 % in BMM started self-treatment. Hormonal treatments have medical contraindications such as polycythaemia or thrombosis, among others [26]. Denial of hormone access, whether for medical or social reasons (such as not accomplishing certain gender roles), poses important considerations for the health of trans* individuals, emphasizing the risks involved, especially in taking hormones without initial medical supervisation.

The study reported that a considerable percentage of trans* people in the BMM group did not find psychiatric (76.2 %) and endocrinologic (55 %) attention useful. Some missing answers were attributed to the non-mandatory nature of these questions, indicating that several people did not require specialized care. Additionally, some individuals did not seek any healthcare services. These findings challenge assumptions about the health needs of trans* individuals, suggesting that they may not universally require specialized care beyond the one for general population.

Several participants in both models sought private surgical services, incurring significant personal expenses. Gender affirming surgeries have had a lack of robust, independent research on the quality of surgical care. This emphasizes the lack of knowledge and the need for more research on the benefits and the risks associated to these surgeries. Specially, research that focuses on patient outcomes rather than surgeons' perspectives [25].

The study found that individuals visiting the BMM center reported heightened feelings of sickness or questioning, with the center frequently recurring traditional gender roles. This suggests that the BMM center upholds to rigid concepts of masculinity and femininity, potentially perpetuating the stigmatization of trans* individuals. Trans* health research has traditionally focused on talking *about* trans* people rather than *with* them. Consequently, these communities have had limited involvement in research [25].

It is important to highlight that the study's objective is to compare the BMM and BPSM models rather than evaluate healthcare workers. While empathy ratings for healthcare workers in BMM were scattered compared to BPSM, it is crucial to note that this difference is likely a result of the model's direct targeting of the trans* community, rather than indicative of negative attitudes from the healthcare workers themselves.

Some participants reported that BMM center insisted on the need for hysterectomy, based on an “increased risk of cancer” with hormonal therapies, despite there being no scientific evidence to support prophylactic annexectomy with or without hysterectomy for health benefits in trans* individuals [1]. The call for robust studies on these topics is emphasized, cautioning against performing surgeries of this magnitude on a preventive basis without strong scientific evidence.

Furthermore, the study draws attention to the personal and structural barriers faced by trans* individuals when accessing preventive activities, such as cervical cancer screening for those with a uterus. Exclusion from such activities should be reconsidered, given that trans* individuals may be at equal or even higher risk of acquiring human papillomavirus [27]. Further research is needed to identify health risks in the trans* community and determine appropriate preventive measures.

## 5 Limitations and strengths

The study has notable strengths, representing the first formal investigation into the impact of trans* healthcare centers on their mental health, while protecting and promoting a vision of trans* health through an inclusive and depathologizing perspective.

However, there are several limitations to acknowledge. The inability to ascertain the number of participants reached hinders the estimation of completion or view rates, which would have enhanced survey reliability. Despite efforts to create a specialized survey for trans* youth, no responses were obtained, creating a significant gap in representation for this population. The sampling method involved LGTBIQ+ organizations in Catalonia and participants from Transit and the GIU at Clinic Hospital. Consequently, it is important to acknowledge that the study may not have reached trans* individuals outside the organizations or healthcare centers. Future investigations should incorporate a substantial sample size to enhance statistical inference.

Moreover, potential bias in responses should be considered, especially as nearly 70 % of people in the BPSM group disagreed that their well-being had deteriorated, while 48 % reported a mental health diagnosis. This discrepancy may be influenced by the social movement for trans* rights, potentially leading to overestimations in evaluations of the Transit Service (BPSM). The study did not explore other potential stressors for mental health outcomes, such as social-demographic variables, leaving room for additional reasons beyond gender identity discrimination for the observed prevalence.

It is crucial to note that the small sample size limits the generalizability of the results, introducing imprecision in external validity and requiring caution in interpretation. It is clear that a study of this nature is crucial, but additional resources are required to reach a broader audience. This would enhance the external validity and the generalizability of the results.

Additionally, the study recognizes the importance of self-criticism. Conducted by cisgender researchers who do not live the trans* realities and experiences, the absence of trans* researchers in the study is acknowledged. However, the study did include the supervision and advice of trans* individuals, providing valuable input.

## 6 Conclusions

The trans* community has tirelessly advocated for aknowledgement in society, confronting the notion that gender diversity is a pathology or that dissident corporealities are a disease. Their resilience, demonstrated through organization and activism, rejects the label of a “victimized marginalized group” [25]. Despite these efforts, negative health effects of stigma and discrimination persist, is evident in the high rates of mental health diagnoses reported in the study. Transphobia, in particular, has been linked to increased anxiety, depression, suicidal ideation, and suicidal intention, and there has been no shortage of news about transgender suicide [[11], [14], [15], [17]].

The study highlights the need for healthcare centers to facilitate access for the trans* community, urging a shift from paternalistic medical roles to informed decision-making and progressive autonomy. Achieving this requires extensive scientific research into the health inequities experienced by trans* individuals. More importantly, the study emphasizes the necessity for the medical community to listen to trans* community voices and actively involve trans* researchers in the production of knowledge.

The lack of positive trans* role models has historically hindered the full embrace of gender identity, as trans* people may not feel reflected in the stereotypical models. Nevertheless, the increased visibility of trans* people and queer political movements have initiated a positive shift in this paradigm.

Recent changes in Spanish legislation emphasize the imperative to protect and promote the health of trans* individuals. Article 56 of the new trans* law highlights "health attention to trans people will be realised according to no pathologization, autonomy, decision and informed consent principles" [28]. However, it is crucial to acknowledge that certain gender identities, such as non-binary, are still not officially recognized, indicating ongoing work ahead.

In conclusion, the study highlights the significance of patient-centered care and sensitivity to dissident gender identities in healthcare settings, advocating for the creation of welcoming and inclusive environments. Perceiving the trans* experience as an individual disease, rather than an effect of societal norms on dissident bodies, has detrimental effects for the trans* community. The call for more scientific research to address the specific needs of the trans* community is echoed, with a strong emphasis on including trans* researchers in the scientific production of knowledge.

## Ethics declaration

The study protocol, encompassing all recruitment methods, broadcast messages, and the survey itself, received approval from the Parc de Salut Mar Research Ethics Committee (file nº 2022/10619). To ensure informed consent, a mandatory question was incorporated at the survey's outset. The survey included contact details, including an email address and webpage link, for participants to address any questions or concerns. The collected information was anonymized, rendering personal identification of participants impossible. No incentives were provided for participation. Participants were required to be 16 years old and above, considering the age limit at which parental consent is not obligatory according to Spanish law.

## Founding statement

This study was supported by the Vice-rectorate for Research Promotion of the 10.13039/501100005774University of Barcelona.

## Data availability

The data associated with the present study, which is included in the manuscript, has not been deposited into a publicly available repository.

## CRediT authorship contribution statement

**Maria Presague-Peciña:** Writing – original draft, Methodology, Investigation, Formal analysis, Conceptualization. **Pepita Giménez-Bonafé:** Writing – review & editing, Supervision, Resources, Investigation, Funding acquisition, Formal analysis, Conceptualization.

## Declaration of competing interest

The authors declare that they have no known competing financial interests or personal relationships that could have appeared to influence the work reported in this paper.

## Appendixes

### ***Appendix I.****Historical framework of trans* healthcare models in Spain*

Discrimination and violence against the trans* community have persisted since they openly expressed their dissident identities and gender expressions.

In 1966, Harry Benjamin published “The Transsexual Phenomenon,” laying the groundwork for the biomedical model of healthcare for transsexual people, then known as those who rejected the gender assigned at birth [29]. In 1978, the Harry Benjamin International Gender Dysphoria Association for Transgender Health was established, now known as the World Professional Association for Transgender Health (WPATH) [11,30]. However, this turning point marked the beginning of neglecting the transgender community's demands, such as legal recognition of their identity or the legalization of hormonal and surgical interventions. Consequently, the paradigm of “transsexuality treatment” was established [9,11].

The inclusion of the transsexual diagnosis as a mental health disorder first occurred in 1975 in the 9th edition of the International Classification of Diseases (ICD-9) [3,11]. Subsequently, in 1980, the third edition of the Diagnostic and Statistical Manual of Mental Disorders (DSM-III) also included this classification. These events pathologized the trans* community, subjecting them to social stigma and discrimination. However, it is essential to note that just a few years earlier, in 1973, homosexuality was partially removed from the DSM-II and completely eliminated from DSM-III in 1978 [14].

In 1979, WPATH published its initial guidelines (Standards of Care first edition), establishing the gold standard for the biomedical model of trans* healthcare. These guidelines, advocating a biological basis (then called Harry Benjamin Syndrome), posited that this psychiatric condition should be treated and corrected [9,11]. The most recent version of the Standards of Care, the eighth edition, was published in 2022 [31].

In post-dictatorship Spain, the LGTB community (LGTB stands for lesbians, gays, trans, bisexuals, intersexual, and queer people) began to self-organize and occupy public spaces. Concurrently, LGTB rights were gaining recognition as human rights across Europe, necessitating Spain's acknowledgment to align itself with social democracy [11].

During the 1980s and 1990s, there was a growing tolerance and freedom for marginalized communities. Homosexuality was decriminalized in Spain in 1979, and a year later, gay and lesbian associations were legalized. In 1983, the Penal Code underwent reform, and sex reassignment surgeries were no longer categorized as castration, allowing the first vaginoplasty in private healthcare [11]. Prior to this change, only a few trans* individuals who could afford it had to travel to Morocco to pursue this goal [32].

Despite the improvements in the Penal Code in 1983, the Royal Decree 63/1995 on the organization of health services in the National Health System explicitly rejected such surgeries, “except the repairing one in pathological intersexual states” [33]. This was a significant setback for the trans* community [9].

Despite legal barriers, a group of doctors in public hospitals began providing medical care to transgender people following the Penal Code reform. Although national-level public funding for these treatments was denied, the decentralized structure in the Autonomous Communities allowed some changes. In 1998, the Andalusian parliament became the first to approve hormonal and surgical treatment for transgender people within the public health system. A year later, the Gender Identity Disorder Unit, now called Gender Identity Units (GIU), was established at Carlos Haya Hospital. This move positioned Andalusia as the first region to adopt European recommendations, incorporating “integral treatment of sex reassignment” into its health benefits [9]. In Catalonia, the Clinic Hospital in Barcelona started offering support to transgender people in line with the Standards of Care guidelines in 2008 [11,30]. Subsequently, various court rulings, including the cases of Jamie in Valencia and Katia in Madrid, compelled other Autonomous Communities to cover surgical treatments [9].

The diverse Gender Identity Units (GIUs) established in Spain were consolidated into a multidisciplinary work group known as the Identity and Sexual Differentiation Group, promoted by the Spanish Society of Endocrinology and Nutrition (GIDSEEN). This group focused on advancing education and research and formulated the Clinical Practice Guidelines for Assessment and Treatment of Transsexualism. These guidelines became the standard reference for trans* healthcare in Spain, aligning with the Biomedical model [8].

However, the trans* community was not involved in the development of these protocols. Since their publication, the community has criticized them for being pathologizing and stigmatizing. These guidelines adhere to traditional gender roles and stereotypes, lacking a comprehensive understanding of the diversity within the community, including various forms and rhythms of transition. Consequently, individuals who did not conform to these roles were excluded from receiving specialized healthcare [14].

In Catalonia, the depathologization movement emerged in 2006 with the Guerrilla Travolaka group. In their manifesto, they stated: “We question the Spanish medical protocol that for years has established absurd and transphobic guidelines for any citizen who wishes to take hormones of their opposite ‘sex.’ We do not believe in gender dysphoria or identity disorders; we do not believe in the madness of the people but in the madness of the system. We do not classify ourselves by sex; we are all different regardless of our genitals, our hormones, our lips, eyes, hands …." [34]. Subsequently, various organizations were established, including Trans Block (2009), Trans*feminist Assembly (2010), and the Stop Trans Pathologization platform (2009), among others. The latter platform initiated one of the most influential depathologization campaigns called STOP Trans Pathologization 2012, International Campaign STP, advocating for a healthcare system that respected the community's rights [14].

The progressive acknowledgment of LGTB rights culminated in the enactment of a law in 2007 that governed the rectification of an individual's sex in the civil registry. To qualify for sex rectification, two criteria had to be met: a medical report diagnosing “gender dysphoria” (Article 4.1.a) and the completion of two years of hormonal therapy (Article 4.1.b) were required [35].

In 2012, gynecologist Rosa Almirall began offering voluntary healthcare to trans* individuals during her “free time” in Barcelona. Almirall viewed the trans* community as an expression of gender diversity rather than a condition to be treated. This innovative approach garnered significant interest, resulting in increased demand, and eventually leading to the establishment of the Transit Service as part of the Catalonia Health Care System. The Transit Service provides healthcare without stigma or judgment and was embraced and accepted by the trans* community. Consequently, it was instituted as a healthcare system with a biopsychosocial approach and an inclusive perspective on health, particularly recognizing the self-determination of gender identity [14].

For years, the trans* community in Catalonia has advocated for recognition and their right to access healthcare without pathologization. In 2014, Law November 2014 was enacted to protect LGTB rights and establish a regulatory framework that respected trans* diversity [13,14]. Article 16th of the law aimed to ensure comprehensive care for trans* individuals on equal terms and encourage community involvement in decision-making [36].

On September 21st, 2016, the *Trans**Forma la Salut platform,* comprising various trans associations, issued a Claim for a new healthcare model for trans* people, urging the implementation of existing regulations (Law 11/2014). The proposed model aimed to offer supportive rather than judgmental care, striving for a comprehensive, respectful, sustainable, public, and free service accessible to all individuals without exclusions. The platform advocated for the recognition of the Transit Service as a reference center due to its commitment to providing respectful healthcare [14].

On November 10th, 2017, the 14/2017 CatSalut (Catalonian Health Care System) Instruction for the implementation of the healthcare model for trans* people in Catalonia came into effect [12]. This marked a significant shift in paradigm, with the Transit Service becoming the primary provider of healthcare to trans* individuals. The GIU at Clinic Hospital assumed a predominant role in the management of surgical treatments.

This historical context establishes the framework for the study.
