# Supplementary material for: Comparative study of trans* healthcare models in Catalonia
Source: Heliyon. 2024 Aug 14;10(18):e36174. doi: 10.1016/j.heliyon.2024.e36174 (PMC11414487; doi:10.1016/j.heliyon.2024.e36174)
Supplement: Multimedia component 1 [file mmc1.docx]

***Supplementary Data.***

***Survey form***

**Section 1.** Introduction

“Hi everyone! We are running a new project to know more about trans* healthcare models!

We are different researchers conducting a study addressed to the trans* community. The main objective is to understand the two models of trans* healthcare that have been a reference in Catalonia and to assess their effects on the psychosocial sphere of people who have sought care.

The survey has been supervised by organizations such as *ACATHI*, *Chrysallis Catalunya*, and *Casal Lambda*, among others. For more information about the study and its foundations, please visit the following website: <https://mariapres21.wixsite.com/estudi-comparatiu-de>

*Data Treatment and Questionnaire Functioning*

The data collected in this study does not allow for personal identification. It is important to note that the form is completely anonymous and voluntary. You can answer the questions you feel comfortable. The estimated time to complete the survey is 5 minutes.

If you have any doubts, you can write us at [maria.presague01@alumni.upf.edu](mailto:maria.presague01@alumni.upf.edu) (you do not need to include personal information in your message).

Thank you very much for your participation! ☺

Note: The asterisk in the word Trans* represents de community of people with gender identities that dissent the established social norm. It includes a wide variety of gender identities and perceptions that go beyond the one assigned at birth, such as transgender, transsexual, crossdressers, genderqueer, travesties, non-binary, agender, among many others.

**THIS FORM CAN ONLY BE ANSWERED ONCE PER PARTICIPANT!**”

**Section 2.** Language

1. Can you select the language with which you are most comfortable with to fill out the survey?
   1. Catalan
   2. Spanish

**Section 3.** General Data

“In this section, we request general data. It is important as many individuals have been prevented from transitioning due to their sexual orientation or age among other causes.

To participate in the questionnaire, you must self-identity as a person within the trans* community (taking into account the diverse set of identities included within the term, as explained above). Additionally, this project is limited in its ability to include individuals who have only accessed trans* services outside of Catalonia.

First and foremost, we require your consent to use the data that you provide in this questionnaire. You will not be asked for personal information such as your name, date of birth or other similar information. Remember that the questionnaire is anonymous and you can leave it at any time.

If you have any doubts about the treatment of data, you can contact us directly at [maria.presague01@alumni.upf.edu](mailto:maria.presague01@alumni.upf.edu). If you want to contact us, you do not need to provide any personal information.”

1. *Informed consent.* Do you consent to using the data we collect in this survey anonymously?
   1. Yes
   2. No (if this option was selected, the form ended)
2. Are you (or have you been) part of the trans* community?
   1. Yes
   2. No (if this option was selected, the form ended)
3. Have you ever accessed any of the following healthcare centres described below?
   1. Hospital Clinic’s Gender Identity Unit or to Transit Service
   2. I have never accessed any of the above centres, but I have accessed other centres outside Catalonia (if this option was selected, the form ended)
   3. I have never accessed any trans* healthcare centre
4. What is your age? If you are under 16 years and would like to participate in the study, please contact us ([maria.presague01@alumni.upf.edu](mailto:maria.presague01@alumni.upf.edu)) to be included to the study through *Crysallis Catalunya*.
   1. < 16 years (if this option was selected, the form went directly to section 7)
   2. 16 – 18 years
   3. 18 – 20 years
   4. 20 – 25 years
   5. 25 – 30 years
   6. 30 – 35 years
   7. 35 – 40 years
   8. 40 – 45 years
   9. 45 – 50 years
   10. 50 – 55 years
   11. 55 – 60 years
   12. 60 – 65 years
   13. > 65 years
5. *Gender Identity.* The concept of gender identity can be understood as a continuum where multiple identities are encompassed. The most extreme zones are accepted as male or female, but the reality is that in the middle, there is a whole spectrum of identities that are often not considered. Please indicate which identity you feel most identified with. If you have not defined it yet, please indicate where you feel most comfortable right now. (the Gender Bread photo was attached to a better understanding, available at <https://www.genderbread.org/>).
   1. Man
   2. Female
   3. Non-binary (not identifying as a man or woman, outside the gender binary)
   4. Gender fluid (not identifying with a single gender identity, but fluctuating between several)
   5. Agender (not identifying with any specific gender identity)
   6. Other:
6. *Gender Expression*. Indicate on a scale from feminine to masculine how you identify your gender expression. The most intermediate zones would be socially understood as androgynous, that is, gender expression that does not follow hegemonic binary roles (the same Gender Bread photo was attached).
   1. Feminine
   2. Approximately feminine
   3. Androgynous
   4. Approximately masculine
   5. Masculine
7. *Biologic sex*. At birth, we are all assigned a gender associated with our biological sex, which is defined by external and internal genitalia, reproductive organs, sex chromosomes, and hormonal levels. Please indicate here which sex was assigned to you at birth (the same Gender Bread photo was attached).
   1. Female
   2. Male
   3. Intersexual
8. *Sexual orientation*. Please indicate here what is your sexual orientation (the same Gender Bread photo was attached). This diagram does not represent asexual people, but they still are included in the responses. This question is important because the quality of care varies among different healthcare centres in relation to sexual orientation.
   1. Heterosexual
   2. Bisexual
   3. Homosexual
   4. Asexual
   5. Others:
9. *Vulnerable sections*. Within the trans* community, there are realities that can sometimes be underrepresented, or other realities that, despite being more common, have added social oppressions to being trans*. Do you belong to any of the following sectors? You can mark it even if you were part of it in the past and not currently (this was a multiple-answer question).
   1. Trans* youth
   2. Trans* elderly
   3. Trans* women
   4. Low socioeconomic level
   5. Trans* people without legal transition
   6. Trans* families (trans* parents)
   7. Trans* migrants
   8. People deprived of liberty
   9. Sex workers
   10. HIV+
   11. Functional diversity
   12. I do not belong to any of the above mentioned
   13. Others:
10. *Mental Health*. Have you ever had a mental health diagnosis? It is not necessary to be an active diagnose.
    1. Depression
    2. Anxiety or anguish
    3. Eating disorders (anorexia, bulimia, among others)
    4. Addictive disorders (alcohol or other drugs)
    5. Attempted suicide
    6. Thoughts of dead
    7. Self-harm
    8. Autism spectrum disorder
    9. Personality disorder
    10. Paranoid disorder
    11. Schizophrenia
    12. Bipolar disorder
    13. Dementia
    14. Obsessive compulsive disorder
    15. Post-traumatic stress disorder
    16. Emotional distress (without any diagnosis)
    17. I did not have any of the above
    18. I did not know
    19. Others:
11. *Psychologic attention*. Have you ever accessed to psychological support? How was this support financed?
    1. Yes, through Public Healthcare
    2. Yes, through support networks provided by LGBTQ+ associations
    3. Yes, through private healthcare
    4. No, I have never accessed psychological support
12. *Personal Well-Being*. It is important to gather participants’ perception of good mental health. You have to respond to the following questionnaire, indicating how often you have experienced the following situations in the last two weeks. This questionnaire has been extracted from the World Health Organization (WHO5 Well-Being Index), which is available at <https://www.psykiatri-regionh.dk/who-5/Documents/WHO5_Spanish.pdf>

| Over las two weeks: | All the time | Most of time | More than half of the time | Less than half of the time | Some of the time | At no time |
| --- | --- | --- | --- | --- | --- | --- |
| I have felt cheerful and in good spirits | 5 | 4 | 3 | 2 | 1 | 0 |
| I have felt calm and relaxed | 5 | 4 | 3 | 2 | 1 | 0 |
| I have felt active and vigorous | 5 | 4 | 3 | 2 | 1 | 0 |
| I woke up feeling fresh and rested | 5 | 4 | 3 | 2 | 1 | 0 |
| My daly life has been filled with things that interest me | 5 | 4 | 3 | 2 | 1 | 0 |

**Section 4.** Clinic Hospital’s Gender Identity Unit

The Clinic Hospital’s Gender Identity Unit (GIU) has been functioning for many years, and until five years ago, it was the reference centre for specialised trans* people healthcare. This section asks about different aspects of the care provided at this centre.

1. Have you ever accessed this service?
   1. Yes, I have also accessed Transit Service
   2. Yes, I have only accessed GIU
   3. No, I have never accessed any trans* healthcare centre (if this option was selected, the form went directly to section 6)
   4. No, but I actually have accessed Transit Service (if this option was selected, the form went directly to section 5)
2. When did you accessed this service for the first time? It can be approximate dates.
   1. Enter date
3. Have you ever engaged in self-treatment (hormonal treatments without medical control)? When did you started it?
   1. Before accessing GIU
   2. During the access to GIU
   3. After accessing GIU
   4. I have never engaged in self-treatment
4. If you have ever performed self-treatment, what was the reason why you started it?
   1. I was denied access to hormonal therapy
   2. Other colleagues had done it with good results, and they advised me
   3. I did not have access to medical care or healthcare centres
   4. I do not know
   5. Others:
5. Have you ever suffered any adverse effects from self-treatment?
   1. Yes
   2. No
6. Was the psychological attention useful in the GIU centre?
   1. Yes
   2. No
7. What aspects of this service would you have improved?
   1. I did not needed this attention
   2. Lack of resources of the unit (needed more hours, individualized sessions, etc.)
   3. My goals and the therapy goals were not the same
   4. I did not obtain the expected results with the therapy
   5. I do not know
   6. Others:
8. Was the endocrinological attention useful in the GIU centre?
   1. Yes
   2. No
9. What aspects of this service would you have improved?
   1. More information related to hormones
   2. More information related to adverse effects
   3. More information related to expected effects
   4. Treatment dosage
   5. I was denied access to hormonal therapy
   6. I do not know
   7. Others:
10. Did you used the surgical services of GIU?
    1. Yes
    2. No, but I will do it in the future
    3. No, and I have no intention of doing so
    4. No, I have already accessed to private surgical services
11. (If the participant accessed to private surgical services) What was the economic cost of the surgery?
    1. Indicate the amount
12. (If the participant accessed to private surgical services) Which country did you undergo the procedure?
    1. Indicate the country
13. During the time you went to GIU, did you suffer any mental health disorder?
    1. Depression
    2. Anxiety
    3. Eating disorders
    4. Addictive disorders
    5. Suicidal attempts
    6. Thoughts of dead
    7. Self-harm
    8. Emotional Distress (without any diagnosis)
    9. None of the above has ever happened to me
    10. I do not know
    11. Others:
14. Did you suffer this affliction before accessing GIU?
    1. Yes, it has happened to me before
    2. No, it has never happened to me before
    3. No, it started after accessing GIU
    4. I do not know
    5. Others:
15. Have you ever lied in consultation?
    1. Yes
    2. No
16. *Healthcare evaluation*. Below are a series of statements in which you should indicate whether you are more in disagree or more in agree.

|  | Strongly disagree | Disagree | Neutral | Agree | Strongly agree |
| --- | --- | --- | --- | --- | --- |
| I was respected in my identity construction process |  |  |  |  |  |
| I had a decision-making capacity about the treatment |  |  |  |  |  |
| I felt questioned |  |  |  |  |  |
| I felt sick |  |  |  |  |  |
| My well-being deteriorated |  |  |  |  |  |
| Classical gender roles were used several times |  |  |  |  |  |
| Healthcare workers empathized with me |  |  |  |  |  |

**Section 5.** Transit Service

Transit Service started its activity in 2012 with the aim of providing more personalized care, respecting the diversity of transitions and identities. In this section, we ask about different aspects of this service.

1. Have you ever accessed this service?
   1. Yes
   2. No
2. Which unit did you accessed?
   1. CAP Sant Fèlix (Sabadell)
   2. CAP Numància (Barcelona)
   3. CAP BAges (Manresa)
   4. CAP Rambla Ferran (Lleida)
   5. CAP Sant Pere (Reus)
   6. Centre de Salut Güell (Girona)
3. When did you accessed this service for the first time? It can be approximate dates.
   1. Enter date
4. Have you ever engaged in self-treatment (hormonal treatments without medical control)? When did you started it?
5. Before accessing Transit
6. During the access to Transit
7. After accessing Transit
8. I have never engaged in self-treatment
9. If you have ever performed self-treatment, what was the reason why you started it?
10. I was denied access to hormonal therapy
11. Other colleagues had done it with good results and they advised me
12. I did not have access to medical care or healthcare centres
13. I do not know
14. Others:
15. Have you ever suffered any adverse effects from self-treatment?
16. Yes
17. No
18. Did you used the psychological attention offered in Transit service?
    1. Yes
    2. No
19. Was the psychological attention useful in Transit service?
20. Yes
21. No
22. What aspects of this service would you have improved?
23. I did not needed this attention
24. Lack of resources of the unit (needed more hours, individualized sessions, etc.)
25. My goals and the therapy goals were not the same
26. I did not obtain the expected results with the therapy
27. I do not know
28. Others:
29. Was the endocrinological attention useful in Transit service?
30. Yes
31. No
32. I did not demand it
33. What aspects of this service would you have improved?
34. More information related to hormones
35. More information related to adverse effects
36. More information related to expected effects
37. Treatment dosage
38. I was denied access to hormonal therapy
39. I do not know
40. Others:
41. Did you used the surgical services?
42. Yes
43. No, but I will do it in the future
44. No, and I have no intention of doing so
45. No, I have already accessed to private surgical services
46. (If the participant accessed to private surgical services) What was the economic cost of the surgery?
47. Indicate the amount
48. (If the participant accessed to private surgical services) Which country did you undergo the procedure?
49. Indicate the country
50. During the time you went to Transit, did you suffer any mental health disorder?
51. Depression
52. Anxiety
53. Eating disorders
54. Addictive disorders
55. Suicidal attempts
56. Thoughts of dead
57. Self-harm
58. Emotional Distress (without any diagnosis)
59. None of the above has ever happened to me
60. I do not know
61. Others:
62. Did you suffered this affliction before accessing Transit?
63. Yes, it has happened to me before
64. No, it has never happened to me before
65. No, it started after accessing Transit
66. I do not know
67. Others:
68. Have you ever lied in consultation?
69. Yes
70. No
71. *Healthcare evaluation*. Below are a series of statements in which you should indicate whether you are more in disagree or more in agree.

|  | Strongly disagree | Disagree | Neutral | Agree | Strongly agree |
| --- | --- | --- | --- | --- | --- |
| I was respected in my identity construction process |  |  |  |  |  |
| I had a decision-making capacity about the treatment |  |  |  |  |  |
| I felt questioned |  |  |  |  |  |
| I felt sick |  |  |  |  |  |
| My well-being deteriorated |  |  |  |  |  |
| Classical gender roles were used several times |  |  |  |  |  |
| Healthcare workers empathized with me |  |  |  |  |  |

**Section 6.** Did not accessed any trans* healthcare centre

This section is designed for people who have never needed or wanted to seek care at the two previous healthcare centres. It is important to identify the reasons why people decide not to access these services, which are available to the population.

1. Why you have never accessed the healthcare centres explained before?
2. I did not needed it
3. I have friends who have gone, and it did not go well
4. I did not know they existed
5. I did not had access to public healthcare centres
6. I do not now
7. Others:
8. Have you ever engaged self-treatment (hormonal treatments without medical control)?
   1. Yes
   2. No
9. If you have ever performed self-treatment, what was the reason why you started it?
10. I was denied access to hormonal therapy
11. Other colleagues had done it with good results and they advised me
12. I did not have access to medical care or healthcare centres
13. I do not know
14. Others:
15. Have you ever suffered any adverse effects from self-treatment?
16. Yes
17. No
18. Did you used private surgical services?
    1. Yes
    2. No
19. (If the participant accessed to private surgical services) What was the economic cost of the surgery?
20. Indicate the amount
21. (If the participant accessed to private surgical services) Which country did you undergo the procedure?
22. Indicate the country

**Section 7**. Trans* youth (only for people who answered < 16 years in question number 5)

If you have arrived at this section, it is because you are under 16 years old. To participate in the questionnaire, a review of the content by legal guardians is necessary. In order to participate, it will be done jointly with the *Chrysallis Catalunya* association, using a version of the form adapted for youth.

If you are still interested in being part of the project, do not hesitate to contact us at [maria.resague01@alumni.upf.edu](mailto:maria.resague01@alumni.upf.edu), and we will make it possible.

Thank you very much for understanding! ☺

**Section 8**. Final valuation (this section was for self-criticism and evaluation of the survey)

This question provides a space for reflection, information, experiences or opinions that anyone would like to share anonymously. It will not be part of the study.
